# Racial and Treatment Center Differences on Time to Treatment Initiation for Nonsmall Cell Lung Cancer Patients Receiving Radiation Therapy As an Initial Treatment

**DOI:** 10.1089/heq.2022.0104

**Published:** 2022-08-18

**Authors:** Akhil Rekulapelli, Raj P. Desai, Aditya Narayan, Linda W. Martin, Richard Hall, James M. Larner, Rajesh Balkrishnan

**Affiliations:** University of Virginia School of Medicine, Charlottesville, Virginia, USA.

**Keywords:** lung cancer, time to treatment, racial disparities, treatment facilities, United States

## Abstract

**Objective:**

Because time to treatment has been shown to be associated with increase in the risk of death for Non Small Cell Lung Cancer (NSCLC) patients, we examined the prevalence and magnitude of racial disparities in mean time to radiation therapy (TTRT) for Stage I-III non-small cell lung cancer patients across a variety of treatment facilities.

**Methods:**

Utilizing the United States National Cancer Database (NCDB), we determined differences in TTRT between different races and different treatment facilities.

**Results:**

Concordant with past research, we found that non-White patients and patients treated at academic facilities, regardless of race, have longer mean TTRT, and that racial disparities in TTRT extend across all treatment facilities (all *p*<0.05).

**Conclusions:**

These findings shed light on the potential presence of and impact of structural racism on patients seeking cancer treatment, and the need for further investigation behind the reasonings behind longer TTI for non-White patients. To elucidate the real-world applicability of these results, further investigation into the societal determinants that perpetuate disparity in time to radiation therapy, and potential interventions in the clinical setting to improve cultural and racial sensitivity among healthcare professionals is recommended.

## Introduction

Inequities in health care propagated by structural racism in the United States have been widely discussed and documented through myriad methods. For example, residential segregation, which manifests through neighborhoods with historically large proportions of minority individuals suffering from poverty, low socioeconomic mobility, and home equity, has been shown to negatively impact the outcomes of Black patients receiving treatment for prostate and colorectal cancer.^1,2^

Beyond residential segregation, mortgage lending discrimination, police brutality, and discriminatory land use policies are all facets of structural racism that can negatively impact health outcomes.^3^ However, although it is crucial to evaluate the impacts of structural racism at the community level, it is similarly essential to evaluate these impacts within health care settings. One key method of evaluation is the analysis of time to treatment initiation (TTI), which can provide insight into racial disparities that exist as patients transition from initial evaluations to treatment design and implementation.

TTI is often associated with treatment for cancers, given the prevailing notion is that decreased TTI correlates with improved overall survival for cancer patients.^4–6^ Nonsmall cell lung cancer (NSCLC), specifically, is the most common type of lung cancer diagnosed in the United States, accounting for >85% of lung cancer cases.^7^ A week-long increase in time to treatment has been shown to be associated with a 3.2% and 1.6% increase in the risk of death of stage I and stage II NSCLC patients, respectively.^4^ Furthermore, Latinx and Black NSCLC patients have been found to have longer overall TTI than White patients, raising the question of how access to treatment can be improved for certain patient populations.^5,8^

Moreover, NSCLC patients treated at academic cancer centers (defined by the Commission on Cancer as facilities with postgraduate medical education for ≥4 programs, >500 new cancer diagnoses per year, a full scope of cancer diagnostic and treatment services, and participation in cancer-related clinical research through clinical trials or referrals to other facilities) have been found to have longer TTI than patients treated at other facilities.^9,10^

Radiation therapy (RT) is commonly employed in the treatment of NSCLC. Stereotactic body radiation therapy (SBRT) is often used to treat NSCLC that is not amenable to surgical resection, and long-term outcomes of SBRT are close to those of surgical resection.^11,12^ For patients with node-positive NSCLC, concurrent radiation therapy (CRT) is commonly used in patients with unresectable disease. In stage III NSCLC, in an inoperable population, the addition of durvalumab after CRT led to significantly longer survival, and a recent landmark analysis showed 42% of patients treated with CRT followed by durvalumab were alive at 5 years.^13,14^ Delivery of SBRT or CRT requires significant treatment planning and coordination and is arguably among the more complicated treatment modalities in cancer care.

Given the results of past studies investigating TTI for cancer patients, we hypothesized that non-White patients receiving treatment would have longer time to radiation therapy (TTRT) than White patients regardless of treatment center, and that patients at academic hospitals would have the longest TTRT regardless of race relative to patients treated at other treatment venues.^4,8,10^

## Methods

This study protocol was reviewed by the University of Virginia Human Subjects Board and declared as exempt. We screened the United States National Cancer Database (NCDB) for patients diagnosed with primary stage I–III NSCLC between 2004 and 2016 (*N*=623,837). Patients with stage IV cancer were excluded from the study due to the advanced nature of the disease and diverse set of treatment options depending on the sites of metastasis.^15^ We filtered the existing set of patients based on whether the patients were diagnosed and received treatment in the same facility (*N*=575,782). We then selected patients who received treatment at an academic or research facility, a comprehensive community cancer center, community cancer center, or integrated network program (*N*=571,943).

Next, we filtered the existing data set for patients who received RT as their initial treatment (*N*=229,368) and excluded patients who had a TTRT of >365 days due to concerns regarding miscoding, as their records were unlikely to reflect RT as a true frontline therapy (*N*=228,294), and patients who had missing information (*N*=222,715). Utilizing the ANOVA procedure, the differences in mean TTRT were determined between patients of the same race treated at varying treatment facilities. Moreover, utilizing a linear regression, predictors of delays in TTRT were evaluated. All analyses were performed using SAS software, version 9.4 (SAS Institute, Inc., Cary, NC).

## Results

### Patient characteristics

Our study included 222,715 patients treated with NSCLC treated with RT (Table 1). The median age at diagnosis was 68 years, and female patients comprised 45% of our cohort. Black and Latinx patients comprised 12% and 2.1%, respectively. The majority of patients in our study had stage III NSCLC (62%) with the remainder having stage II (13%) and stage I (24%). A significantly greater percentage of patients treated at an academic center were Black (17%) compared with those treated at nonacademic centers (10%), (*p*<0.0001).

**Table 1.** Patient Demographics

Characteristics (***N***=222,715)	Group 1: community (***n***=23,652) ***N*** (%)	Group 2: comprehensive community (***n***=98,998) ***N*** (%)	Group 3: academic (***n***=70,101) ***N*** (%)	Group 4: other (***n***=29,964) ***N*** (%)	***p*-**(chi-square)
Patient characteristics					
Age, years					
40–49	1093 (5)	3994 (4)	3645 (5)	1294 (4)	<0.0001
50–59	4310 (18)	16,483 (17)	13,788 (20)	5170 (17)	
60–69	7700 (33)	30,993 (31	22,370 (32)	9063 (30)	
≥70	10,549 (45)	47,528 (48)	30,298 (43)	14,437 (48)	
Gender					
Male	13,542 (57)	54,577 (55)	38,167 (54)	15,980 (53)	<0.0001
Female	10,200 (43)	44,421 (45)	31,934 (46)	13,984 (47)	
Race					
White	20,898 (88)	87,748 (89)	55,112 (79)	25,378 (85)	<0.0001
Black	2044 (9)	9121 (9)	11,857 (19)	3788 (13)	
Asian	400 (2)	1077 (1)	1594 (2)	376 (1)	
Other	310 (1)	1052 (1)	1538 (2)	422 (1)	
Ethnicity					
Non-Latinx	21,890 (93)	90,831 (92)	64,409 (92)	27,103 (90)	<0.0001
Latinx	395 (2)	1684 (2)	1903 (3)	754 (3)	
Unknown	1367 (6)	6483 (7)	3789 (5)	2107 (7)	
Insurance status					
Medicaid	1921 (8)	5498 (6)	5668 (8)	1920 (6)	<0.0001
Medicare, age <65 years	1863 (6)	6428 (6)	4280 (6)	1842 (6)	
Medicare, age >65 years	12,601 (53)	55,286 (56)	33,067 (47)	16,749 (56)	
Uninsured	676 (3)	2788 (3)	2416 (3)	709 (2)	
Other government	696 (3)	3362 (3)	5222 (7)	810 (3)	
Private	5895 (25)	25,636 (26)	19,448 (28)	7934 (26)	
Population without high school diploma, %					
<7.0	2590 (11)	17,124 (17)	14,400 (21)	6510 (22)	<0.0001
7.0–12.9	7577 (32)	32,511 (33)	21,822 (31)	10,828 (36)	
13.0–20.9	8112 (34)	30,784 (31)	19,788 (28)	8141 (27)	
≥21.0	5373 (23)	18,579 (19)	14,091 (20)	4485 (15)	
Median income, $					
<38,000	5806 (25)	22,238 (22)	15,977 (23)	5780 (19)	<0.0001
38,000–47,999	7937 (34)	28,313 (29)	16,106 (23)	7102 (24)	
48,000–62,999	5934 (25)	26,433 (27)	17,890 (26)	8764 (29)	
≥63,000	3975 (17)	22,014 (22)	20,128 (29)	8318 (28)	
County type					
Urban	22,550 (95)	95,995 (97)	68,967 (98)	29,694 (99)	<0.0001
Rural	1102 (5)	3003 (3)	1134 (2)	270 (1)	
Distance to hospital, median, miles	7.6	9.4	11.2	7.7	<0.0001
Charlson/Deyo comorbidity score					
0	13,912 (59)	55,770 (56)	43,502 (62)	16,899 (56)	<0.0001
1	64,29 (27)	28,438 (29)	17,175 (25)	8431 (28)	
≥2	3311 (14)	14,790 (15)	9424 (13)	4634 (15)	
Cancer identification					
Year of diagnosis					
2004	1801 (8)	7171 (7)	4369 (6)	2050 (7)	
2005	1826 (8)	7337 (7)	4427 (6)	2040 (7)	
2006	1824 (8)	7327 (7)	4529 (6)	2096 (7)	
2007	1836 (8)	7322 (7)	4510 (6)	2158 (7)	
2008	1906 (8)	7509 (8)	4759 (7)	2346 (8)	
2009	1797 (8)	7577 (8)	5138 (7)	2318 (8)	
2010	1702 (7)	7130 (7)	5240 (7)	2226 (7)	
2011	1729 (7)	7465 (8)	5587 (8)	2316 (8)	
2012	1735 (7)	7313 (7)	5913 (8)	2402 (8)	
2013	1844 (8)	7875 (8)	6119 (9)	2402 (8)	
2014	1831 (8)	8167 (8)	6229 (9)	2546 (9)	
2015	1924 (8)	8484 (9)	6638 (9)	2583 (9)	
2016	1897 (8)	8321 (8)	6643 (9)	2481 (8)	
Behavior					
*In situ*	5 (0)	10 (0)	8 (0)	3 (0)	0.5518
Invasive	23,647 (100)	98,988 (100)	70,093 (100)	29,961 (100)	
Stage of disease					
I	3992 (17)	23,552 (24)	19,126 (27)	7581 (25)	<0.0001
II	3518 (15)	13,675 (14)	8393 (12)	3940 (13)	
III	16,142 (68)	61,770 (62)	42,852 (61)	18,443 (62)	
Facility location^a^					
Northeast	3435 (15)	15,455 (16)	19703 (28)	4818 (16)	<0.0001
Midwest	8904 (38)	43,779 (44)	23,088 (33)	12,151 (41)	
South	8697 (37)	26,230 (27)	21,466 (31)	9326 (31)	
West	2616 (11)	13,534 (14)	5844 (8)	3669 (12)	

^a^ Regions: northeast (CT, MA, ME, NH, RI, VT, NJ, NY, PA); south (DC, DE, FL, GA, MD, NC, SC, VA, WV, AL, KY, MS, TN, AR, LA, OK, TX); midwest (IL, IN, MI, OH, WI, IA, KS, MN, MO, ND, NE, SD); west (AZ, CO, ID, MT, NM, NV, UT, WY, AK, CA, HI, OR, WA)

### Evaluation of differences in mean TTRT between groups

The mean TTRT for the patient cohort was 61.7 days (confidence interval [95% CI]: 61.6–61.9), whereas for White patients it was 60.9 days (95% CI: 60.7–61.2), that for Black patients was 65.9 days (95% CI: 65.2–66.6), and that for Asian patients was 71.9 days (95% CI: 69.8–73.9). Among White patients, patients treated in community centers had the shortest mean TTRT (57.7 days, 95% CI: 57.1–58.4), whereas patients treated in academic centers had the longest mean TTRT (65.4 days, 95% CI: 61.0–62.3).

Furthermore, among Black patients, patients treated in comprehensive community facilities had the shortest mean TTRT (59.8 days, 95% CI: 58.7–60.9) and patients treated in academic facilities had the longest mean TTRT (71.4 days, 95% CI: 70.4–72.5). Among Asian patients, patients treated in community centers had the shortest mean TTRT (64.8 days, 95% CI: 58.7–71.0), whereas patients treated in academic facilities had the highest mean TTRT (74 days, 95% CI: 72.8–78.9) (Table 2).

**Table 2.** Mean Time to Radiation Therapy for Patients of Varying Race and by Type of Treatment Center

	Group 1: community mean (in days), 95% CI	Group 2: comprehensive community mean (in days), 95% CI	Group 3: academic mean (in days), 95% CI	Group 4: other mean (in days), 95% CI	***p*** ^a^
White	57.7 (57.1–58.4)	58.7 (58.3–59.0)	65.4 (65.0–65.9)	61.6 (61.0–62.3)	<0.0001
Black	64.0 (61.7–66.3)	59.8 (58.7–60.9)	71.4 (70.4–72.5)	64.3 (62.5–66.1)	<0.0001
Asian	64.8 (58.7–71.0)	69.8 (66.3–73.3)	75.8 (72.8–78.9)	68.3 (62.4–74.3)	0.0022
Other	63.2 (57.1–69.2)	62.0 (58.6–65.4)	65.3 (62.5–68.0)	60.5 (55.6–65.3)	0.3015

^a^ *p* Values are for differences in the time to treatment initiation (mean) for each variable. *p* Values are for differences in the median time to treatment initiation for each variable and were calculated using the Kruskal–Wallis test.

CI, confidence interval.

There was a significant difference in mean TTRT among White patients (*p*<0.0001), Black patients (*p*<0.0001), and Asian patients (*p*=0.0002) treated at different facilities. Notably, across all races, patients treated at academic centers had the longest mean TTRT. Finally, across all treatment facilities, except for academic facilities, Asian patients had the longest mean TTRT, with the disparity being the greatest within comprehensive community settings. In comprehensive community facilities, Asian patients had a mean TTRT of 69.8 days, whereas White patients had a mean TTRT of 58.7 days and Black patients had a mean TTRT of 59.8 days.

### Analysis of variable-specific delays in TTRT

Table 3 outlines day-specific disparities in TTRT determined using a linear regression. Black patients had a 5.2-day delay in TTRT compared with White patients (*p*<0.0001, 95% CI: 4.5–5.9), whereas Asian patients had a 7.2-day delay in TTRT compared with White patients (*p*<0.0001, 95% CI: 5.4–8.9). Furthermore, Hispanic patients were found to have a 7.3-day delay in TTRT compared with non-Hispanic patients (*p*<0.0001, 95% CI: 5.8–8.8).

**Table 3.** Predictors of Delay in Time to Radiation Therapy Using a Linear Regression Model

Characteristics	Difference in TTRT mean (in days), 95% CI	***p*** ^a^
Facility type		
Comprehensive community	Reference	—
Community	0.7 (−0.1 to 1.4)	0.08
Academic	6.1 (5.6 to 6.6)	<0.0001
Other	2.8 (2.1 to 3.5)	<0.0001
Age (years)		
40–49	Reference	—
50–59	2.0 (0.8 to 3.2)	0.0005
60–69	3.7 (2.6 to 4.9)	<0.0001
70+	−0.02 (−1.2 to 1.2)	0.97
Gender		
Male	Reference	—
Female	2.4 (1.9 to 2.8)	<0.0001
Race		
White	Reference	
Black	5.2 (4.5 to 5.9)	<0.0001
Asian	7.2 (5.4 to 8.9)	<0.0001
Other		
Hispanic vs. Non-Hispanic		
Non-Hispanic	Reference	
Hispanic	7.3 (5.8 to 8.8)	<0.0001

Variables significant in the ANOVA of all characteristics were included in the multivariate linear regression.

^a^ *p* Values are for mean differences in the time to treatment initiation from the reference value for each variable.

TTRT, time to radiation therapy.

Across treatment settings, compared with patients in comprehensive community settings, patients treated in community treatment settings had no delay in TTRT (*p*=0.08, 95% CI: −0.1 to 1.4), patients in academic settings had a 6.1-day delay in TTRT (*p*<0.0001, 95% CI: 5.6–6.6), and patients in other treatment venues had a 2.8-day delay in TTRT (*p*<0.0001, 95% CI: 2.1–3.5).

## Discussion

This study finds strong associations that suggest non-White patients and patients treated at academic facilities, regardless of race, have longer mean TTRT, and that racial disparities in TTRT extend across all treatment facilities. We believe there are myriad factors behind this demonstrated racial disparity in TTRT, including that non-White patients are more likely to be uninsured or have nonprivate health insurance, face greater socioeconomic barriers when seeking care, experience less satisfaction with physician–patient encounters, and be perceived by providers as at risk for noncompliance.^16,17^

In addition, we cannot fully discern why there are treatment delays for patients treated in academic centers compared with those in community settings. One widely suggested hypothesis is that patients seen in academic centers started out in smaller community facilities and were referred sometime during their workup due to complexity of the case, rather than receiving all workup at one facility. This transfer from a community setting to an academic center is often accompanied by delays in referrals and long waiting lists for appointments.^18^

Although it has been previously recognized that racial disparities in TTRT for NSCLC patients exist, our data demonstrate that disparities exist across all treatment facilities.^5,8^ Within each indicated treatment facility (community, comprehensive community, and academic), White patients had the shortest mean TTRT. Compared with White and Black patients, Asian patients had the longest mean TTRT regardless of treatment facility. Although the representation in health care and socioeconomic status of Asian Americans is disproportionately high compared with their counterparts, the sheer diversity of the racial group still makes health care delivery a challenge.

For example, in a 2021 study of eight Asian American subgroups, stark differences in the average socioeconomic status and proportion of immigrants within each group were found, leading to variability in their self-reported health. Moreover, many native languages of Asian American individuals are only spoken by a small minority of the American population, so for individuals with limited English proficiency, finding effective translation services is a key barrier to attaining treatment.^19^

Although the observed delays in TTRT were limited to a maximum of 7.2 days between patients of different races, this approximately week-long increase in time to treatment has been associated with a 3.2% and 1.6% increase in the risk of death of stage I and stage II NSCLC patients.^4^ Further investigation into the impact of increased TTRT on patient survival is needed to determine the clinical impacts of the observed delays more precisely. In addition, our study is limited by the inability of the NCDB to provide more information about variables such as income, distance from the point of treatment, education, and other socioeconomic factors that potentially confound or otherwise support our results.

Strengths of our study include the use of the NCDB, which accounts for ∼70% of all newly diagnosed cancer cases in the United States through data acquired from >1500 programs accredited by the Commission on Cancer.

Our results suggest that racial disparities in TTRT, wherein non-White patients have been demonstrated to have delayed treatment compared with White patients, are not limited to a particular type of treatment facility. These findings shed light on the potential presence of and impact of structural racism on patients seeking cancer treatment, and the need for further investigation behind the reasonings behind longer TTI for non-White patients.

For example, Black oncology patients have been found to be less likely to have their pain assessed or managed, and racial biases have been found to underly oncological clinical interactions and treatment decisions.^20,21^ It is important to note here that race here is not a predisposing effect, rather a composite of perceived everyday racism (individual level) and residential segregation (neighborhood level) effects that lead to suboptimal care for NSCLC in non-White patients.^21^

Our findings emphasize the need for further investigation into the systematic drivers of racial inequity in access to and quality of cancer care, ranging from the increased representation of certain ethnic groups in medicine to the development of more robust translation services that encompass the hundreds of languages spoken in the United States. In addition, in line with past research into treatment time disparities among various treatment facility types, our study demonstrates that academic medical centers have longer TTRT, suggesting the need for more streamlined patient transitions from community-based to academic center-based medical care.

As a whole, our results shed light on the need for a diverse set of solutions to be implemented in both clinical and nonclinical settings. In specific, further analysis of the deep-rooted impacts of structural racism on the health outcomes of cancer patients seeking treatment is needed to generate wide-reaching efforts to address long-standing inequities in health care. Finally, at the community level, collaboration between providers and community stakeholders and organizations to increase accessibility and patient knowledge of cancer and cancer treatment can help patients understand the need for timely care.^22^

## Abbreviations Used

CI: confidence interval
CRT: concurrent radiation therapy
NCDB: National Cancer Database
NSCLC: nonsmall cell lung cancer
RT: radiation therapy
SBRT: stereotactic body radiation therapy
TTI: time to treatment initiation
TTRT: time to radiation therapy
